# RECURRENT IMPETIGO HERPETIFORMIS WITH DIABETES AND HYPOALBUMINEMIA SUCCESSFULLY TREATED WITH CYCLOSPORINE, ALBUMIN, INSULIN AND METFORMIN

**DOI:** 10.4103/0019-5154.62757

**Published:** 2010

**Authors:** Chembolli Lakshmi, C R Srinivas, Sarah Paul, T V Chitra, K Kanchanamalai, L S Somasundaram

**Affiliations:** *From the Department of Dermatology, Neonatology and Pediatrics, Obstetrics and Gynecology and Medicine, PSG Hospitals, Coimbatore, India.*

**Keywords:** *Impetigo herpetiformis*, *cyclosporine*, *diabetes and hypoalbuminemia*

## Abstract

We report the case of a patient with recurrent impetigo herpetiformis associated with diabetes mellitus, hypoalbuminemia, and hypocalcaemia; who was refractory to corticosteroids. Cyclosporine along with other supportive measures proved to be life-saving with maintenance of pregnancy.

## Introduction

Impetigo herpetiformis (IH) is rare, pustular dermatoses of pregnancy posing serious risk to maternal and fetal well-being.[1] Systemic corticosteroids and multisystemic support constitute the mainstay of therapy. Our patient had recurrent IH associated with fetal loss and presented in her fifth pregnancy with generalized erythema, edema and pustules, metabolic disturbances, hypoalbuminemia, hypocalcemia and signs of toxicity. She was refractory to corticosteroids. Early recognition and initiation of cyclosporine along with insulin and albumin resulted in disease control. Following rupture of membranes in the 36^th^ week, a female baby, large for gestational age (gestational age 35.5 weeks; Wt.3.2 kg; Apgar score 9 at five minutes) was delivered vaginally.

## Case Report

**Figure 1:**
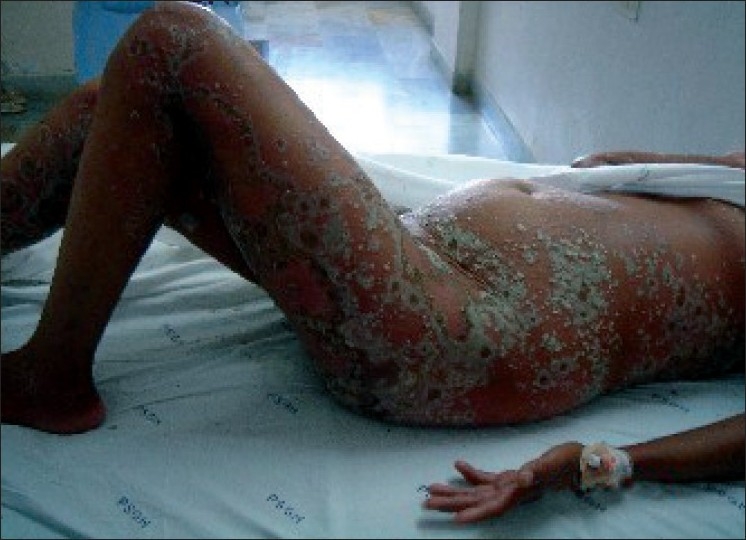
Widespread erythema, edema, and pustules

A 23-year-old female in the 26^th^ week of pregnancy (G_5_P_3_L_1_A_1_), presented with widespread erythema, edema and pustules over the abdomen, arms thighs, legs and buttocks of two weeks duration. [Figure 1]. She had similar lesions during three of her previous pregnancies which had started later around 30-32 weeks. The first was a pre-term delivery and the baby died soon after birth. During the second pregnancy, lesions were present only over the abdomen and she delivered a term live male baby. The third pregnancy resulted in intra-uterine death at term. The fourth pregnancy was terminated medically. There was no previous history of diabetes or hypertension.

**Figure 2:**
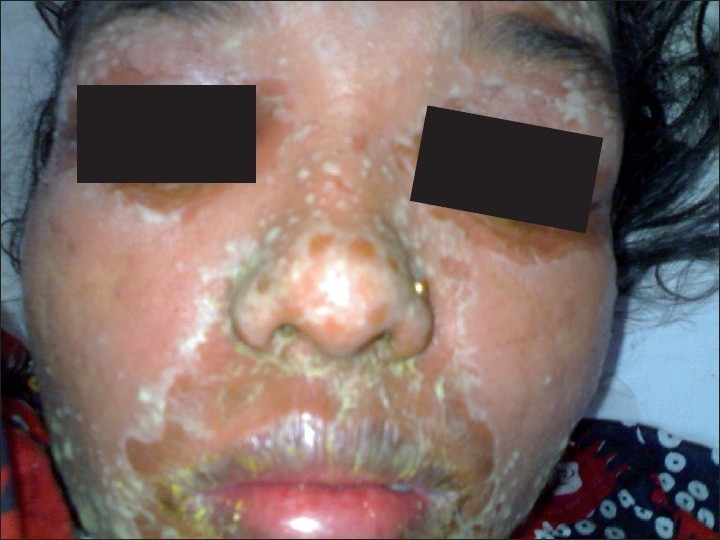
Erythema, edema and coalescent pustules over the face

Clinical examination revealed a toxic, febrile (temperature 39°C) patient with large erythematous, edematous plaques studded with pustules over the abdomen, arms, legs, and buttocks involving about 70% of the body with confluent erythema surrounding the plaques. The erythema and pustulation progressed in waves from the palms and soles to the forearms, abdomen, legs and trunk and finally involved the face and scalp. [Figure 2] The mucosae and eyes were unaffected. Obstetric examination revealed a single live fetus with no anomalies on scanning.

**Figure 3:**
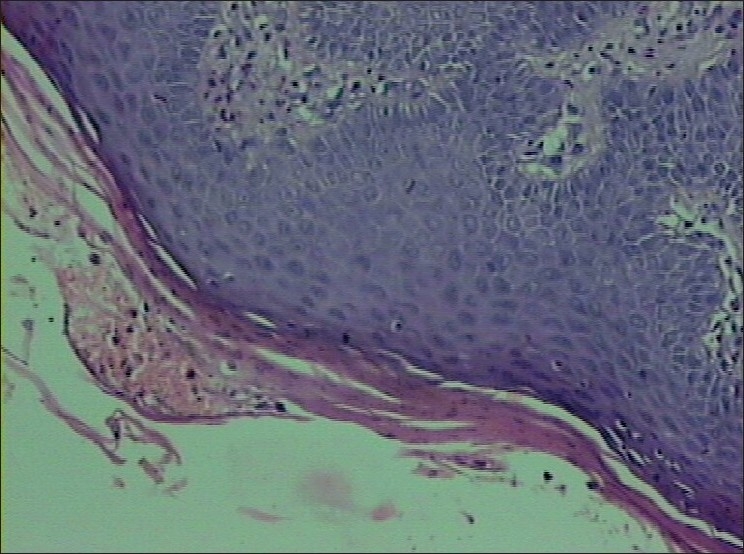
Epidermis shows parakeratosis, subcorneal neutrophilic pustules, acanthosis and regular elongation of rete ridges (H and E stain; magnification ×100)

**Figure 4:**
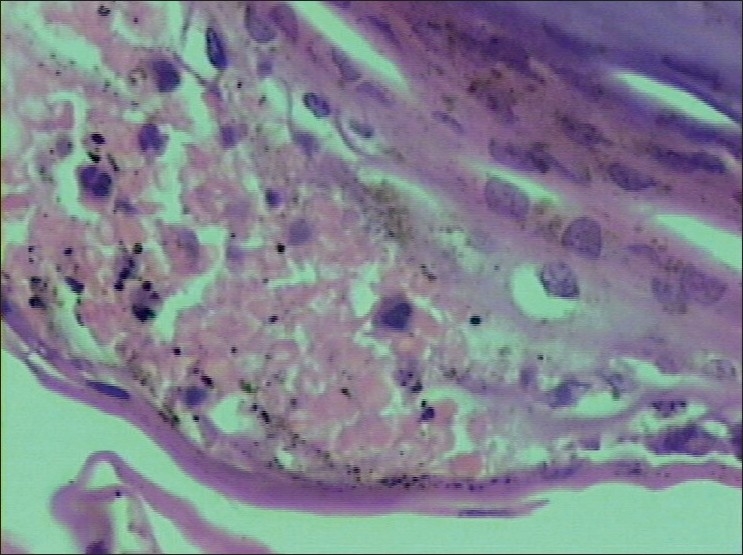
Higher magnification of the subcorneal neutrophilic pustule with overlying parakeratosis (H and E stain; magnification ×400)

Histopathology revealed parakeratosis, subcorneal neutrophilic pustules, acanthosis and regular elongation of rete ridges consistent with IH. [Figures 3 and 4]. Hematological parameters were as follows:

Leukocytosis of 13.3×10^9^ /L with neutrophilia of 85%; hemoglobin of 11.3 g%; a raised erythrocyte sedimentation rate (ESR) of 52 mm/hr; hypoalbuminemia of 3.0 g/dL; fasting blood sugar of 293 mg/dL; post prandial sugar of 512 mg/dL; hypocalcemia of 7.7 mg/dL; glycosylated hemoglobin of 11.5% with ketone bodies in urine of 40 mg/dL. Serum cortisol and thyroid hormone levels were normal. Other liver and renal function tests were normal.

**Figure 5:**
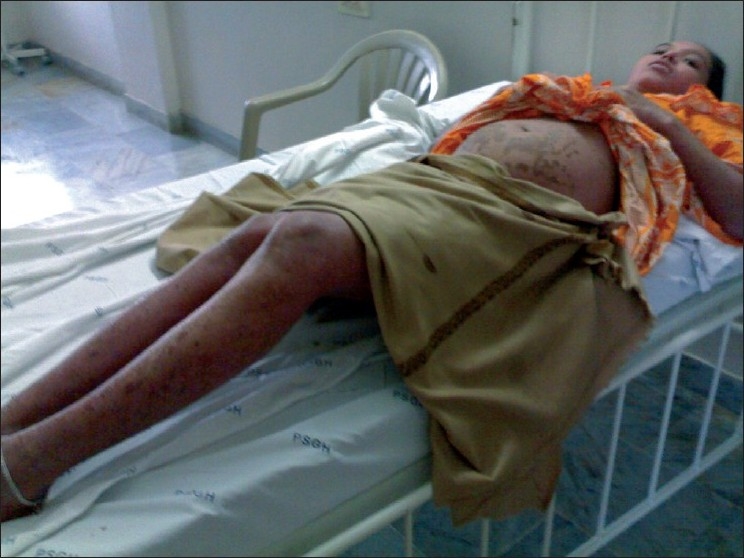
Complete resolution of pustules two weeks after initiating cyclosporine

Prednisolone at a dose of 50 mg (1 mg/kg/d) was initiated along with insulin for 4 days. She was refractory to corticosteroids with worsening of diabetic control and showed disease progression. She was restless and complained of pain over the lesions. Cyclosporine was added at a dose of 150 mg (3 mg/kg/d) and steroids tapered to 20 mg over two weeks. Within three days of initiating cyclosporine, the intense red erythema had acquired a dusky hue but the waves of pustules continued their progression from the legs and hands to the abdomen and trunk and finally involving the face followed by exfoliation. The previous pregnancies were also associated with similar waves subsiding with the involvement of the face. Four days after cyclosporine, she developed pedal edema. Since serum albumin was low, two units of 20% salt free albumin were infused. There was dramatic control of disease progression following the addition of albumin with resolution of pedal edema. [Figure 5]. Her fluctuant blood sugar level was brought under control with insulin (60-0-25) units and later metformin at a dose of 250 mg thrice daily was added. This also helped to suppress her uncontrollable appetite. Potent topical corticosteroids and emollients were used to treat the erythematous and painful lesions over the palms. She was discharged in week 29, free of all lesions. She was followed-up every two weeks and an anomaly scan repeated at every visit (Ultrasound scan in the B mode and M mode to detect congenital anomalies and cardiac anomalies respectively were done). They did not reveal any lethal defects. She developed pustules on the abdomen, neck and legs at 30 weeks so the cyclosporine dose was increased to 200 mg/d.

**Figure 6:**
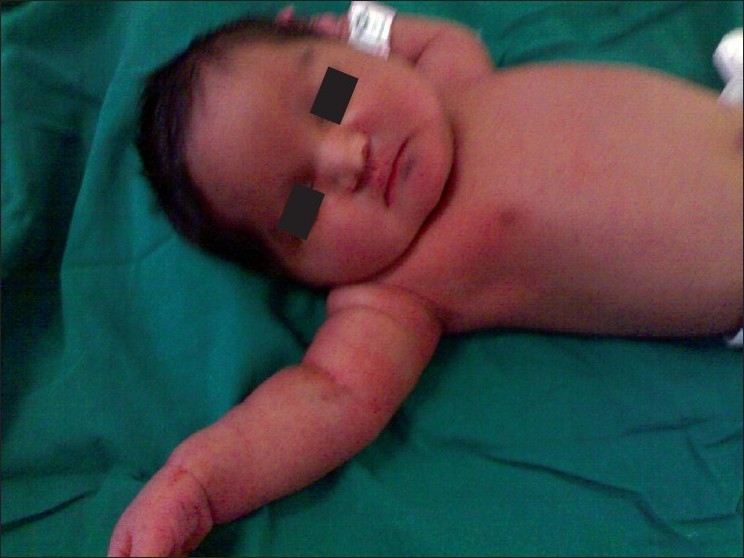
Female baby (35.5 weeks gestational age) showing macrosomy, with chubby facies typical of gestational diabetes three days after delivery

Steroids were further tapered to 10 mg and remained so till delivery. She developed a fresh wave of pustules in week 36, followed by rupture of membranes and vaginal delivery of a female baby with gestational age 36 weeks with an Apgar score of 9 at five minutes. [Figure 6]. The baby had asymptomatic hypoglycemia (Random blood sugar-22 mg/dl), 10% dextrose infusions IV were started (260 ml in 24 hours), and random blood sugar monitored two-hourly. Two days after delivery, nasogastric feeds with Lactogen (formula) were started, 3 ml every three hours; this was increased slowly to 8 ml since the random blood sugar was 59 mg/dL. Sangu feeds with Lactogen were initiated and maintained at 18-20 ml/day. Nasogastric feeds were stopped as soon as the infant tolerated Sangu feeds.

IV dextrose was reduced to 50 ml/24 hrs and stopped prior to discharge. The baby also had polycythemia and developed hyperbilirubinemia (Total bilirubin 6.9 mg/dL; indirect 16.2 mg/dL), which was treated with fresh frozen plasma and phototherapy. Hypocalcemia was also observed (ionized calcium 0.781 mmol/L (Normal- 1.05-1.37). This was treated initially with slow IV infusion of calcium gluconate (3 ml); subsequently the infant was administered Calcium syrup, 2 ml once daily. The sacral area showed a small dimple.

Ultrasound scan of this area revealed a spina bifida occulta. The dermal sinus was not continuous with the spinal canal with conus, cauda equina, nerve roots and spinal canal being normal. The baby did not show any features of growth retardation or immunosuppression. Erythematous papules clinically suggestive of irritant dermatitis developed on the back since the baby was placed on a plastic sheet during phototherapy. This subsided with mild topical corticosteroids. In the mother, the waves of pustules waned the day after delivery with the skin acquiring a dusky hue. Cyclosporine was continued, the dose being tapered to 150 mg one week after delivery, and subsequently tapered by 50 mg every week. She continued to be maintained on insulin injections. Metformin 250 mg thrice daily was restarted five days after delivery since her blood sugar levels showed fluctuation. Since her serum albumin was low (2.3 g/dL), two units of 20% salt-free albumin were infused.

**Figure 7:**
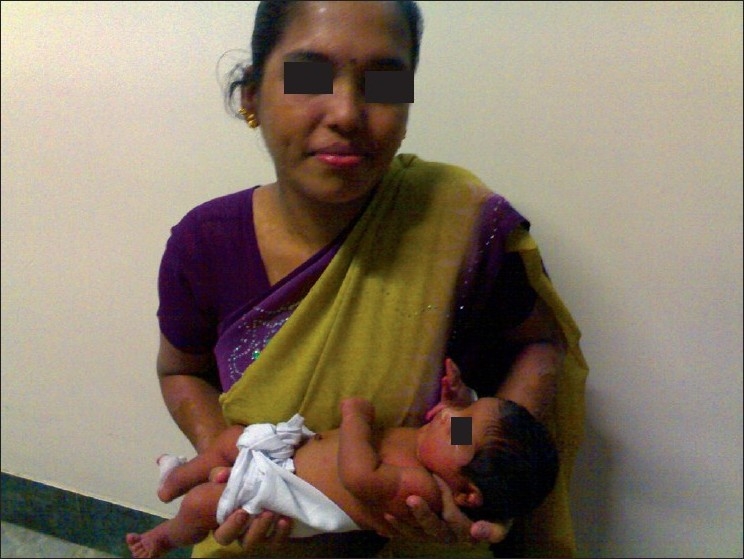
Mother and child at discharge, 10 days after delivery

As noted previously, there was faster resolution of the lesions following the albumin infusion. Breast feeds were withheld while the patient was on cyclosporine and was started three days after withdrawal of cyclosporine. Live viral and bacterial immunization was postponed for the baby. Both the mother and baby continued to do well and were discharged 10 days after delivery [Figure 7]; the baby was on Sangu feeds with Lactogen (20 ml/day). The mother stopped cyclosporine after about two weeks after delivery on her own and started breast feeding three days later along with the formula feeds.

## Discussion

IH is a rare gestational skin disease which poses a high risk for both the mother and the offspring and usually occurs in the last trimester of pregnancy.[1] Recurrent IH is known to occur during subsequent pregnancies with lesions developing earlier.[2,3] The management has traditionally been with systemic corticosteroids but in many patients who prove refractory to corticosteroids and in addition have a metabolic decompensation, cyclosporine may be lifesaving. Few single case reports using cyclosporine for IH were all associated with a good maternal and fetal outcome.[3–5] Complications of IH include placental insufficiency with intrauterine growth retardation and fetal death.[3] Various antenatal and perinatal problems have been discussed in renal transplant patients treated with cyclosporine.[6] Obstetrical examination with anomaly scanning in our patient did not reveal any anomalies or features of growth retardation. However, at birth, the baby had a dimple in the sacral area, which on ultrasound scanning revealed a spina bifida occulta. An MRI scan of the area was recommended three months later to rule out any tethering of the cord. Although initiation of cyclosporine resulted in decrease in the intensity of erythema, the response was more dramatic after 20% salt free albumin was infused with complete resolution of the waves of pustulation during the pregnancy. This response was also observed in the post partum period after infusion of the second unit of albumin.

This report highlights cyclosporine as a life-saving measure in IH refractory to corticosteroids and associated with metabolic disturbances. Correction of associated hypoalbuminemia hastened the resolution of lesions. Spina bifida is one of the commonest congenital anomalies with maternal diabetes posing a risk factor. Our patient had the mildest type, which may not be related to the use of cyclosporine or metformin since neural tube defects occur in the first month of pregnancy.
